# Intolerance of uncertainty and future career anxiety among Chinese undergraduate students during COVID-19 period: Fear of COVID-19 and depression as mediators

**DOI:** 10.3389/fpubh.2022.1015446

**Published:** 2022-11-29

**Authors:** Tianshu Zhou, Yuchang Bao, Danfeng Guo, Yunpeng Bai, Ruizhe Wang, Xinyue Cao, Hebin Li, Yidi Hua

**Affiliations:** ^1^Department of Social and Behavioural Sciences, City University of Hong Kong, Hong Kong, China; ^2^Department of Political Science, Suzhou University of Science and Technology, Suzhou, China; ^3^Faculty of Education, University of Macau, Taipa, China; ^4^Department of Political ScienceSuzhou University of Science and Technology, Suzhou, China; ^5^Department of Applied Social Science, The Hong Kong Polytechnic University, Kowloon, Hong Kong SAR, China; ^6^Department of Media and Communication, Xi'an Jiaotong-Liverpool University, Suzhou, China; ^7^Faculty of Arts, Saint Mary's University, Halifax, NS, Canada

**Keywords:** intolerance of uncertainty, future career anxiety, fear of COVID-19, depression, Chinese undergraduate students

## Abstract

Uncertainty is mushrooming throughout COVID-19, and intolerance of uncertainty (IoU) nudges people into mental health difficulties involving fear, depression, and anxiety. The objective of this study was to investigate the role of depression and fear of COVID-19 (FoC) in the association between IoU and future career anxiety (FCA) among Chinese university students during the COVID-19 pandemic. This study involved 1,919 Chinese undergraduate students from 11 universities in eight Chinese cities with an online self-administered survey that included demographic information, IoU, FoC, depression, and FCA completed by all participants. Our study demonstrated a positive relationship between IoU and FCA and the chain mediation effect of FoC and depression. Thus, understanding how FoC affects FCA not only informs university career professionals and assists students in preparing for employment, but also motivates schools to offer career opportunities workshops and, most importantly, provides mental health support to help students effectively cope with uncertainty and overcome COVID-19-related stress.

## Introduction

The worldwide spectrum of the COVID-19 pandemic that erupted in 2019 is still propagating, which poses an unprecedented public health challenge, with at least 500 million infections and 6 million fatalities projected by May 2022; meanwhile, the world faces economic collapse, with GDP hovering around 3.3% in 2020 (1, 2). The economic spillover effects of unemployment put college students in white-hot competition (3, 4). The equilibrium between supply and demand in China's labor market has been unbalanced, as positions have declined while applicants have surged (5, 6). Chinese students fear for their future job opportunities when COVID-19 dulls the economy's glitter and jacks up the unemployment rate (7). Meanwhile, industrial contraction led to job losses, and rapidly changing hiring requirements fueled career uncertainty.

Although the government's COVID-19 restrictions strategy effectively prevented the spread of the virus, it had a detrimental impact on the mental health of the population, particularly the long-term quarantine. Studies of health workers who were quarantined during the SARS pandemic demonstrated a high level of psychological distress, post-traumatic symptoms, anxiety, and depressive symptoms (8, 9). In addition, studies from the current COVID-19 pandemic support the harmful effects of government restrictions, particularly for college students. In addition to a significant increase in psychological distress, college students also faced financial difficulties, limited supplies, and fears of infection (10, 11). A meta-analysis revealed that approximately 28.4% of college students in China might exhibit depressive symptoms, indicating that depression is prevalent among Chinese college students (12). The pandemic unquestionably worsens the psychological health of students and leads many university students to exhibit depression (11).

Intolerance of uncertainty (IoU) is a personality characteristic in which people perceive unpredictable situations as time bombs threatening their lives (13, 14). When people are aware of uncertainty in their external environment, their anxiety may merge (15–17). Return to life, recurring breakouts and lockdowns, and waves of bankruptcy not only plunge current life into uncertainty but also leave individuals worried about the future (18–20). Lockdown regulation helps predict people's negative emotions because it can happen at any time, making them feel that the world is unpredictable and difficult to control (21). In terms of work, individuals report anxiety and depression when they perceive COVID-19 as a threat to job stability, which is closely connected to survival and social needs (6, 22). When the global economy stays sluggish, Chinese undergraduates were also trapped in an unsteady labor market (23, 24). Whereas, according to Hofstede's dimension theory (25), Chinese culture values certainty, job insecurity is more threatening to those who care about stability (26, 27). Not surprisingly, Chinese students' anxiety about their future careers spikes when they perceive through first-hand experience or social media that COVID-19 disrupts their work plans (5, 28). Moreover, early adulthood is a launching phase for developing job blueprints (29). According to Erikson's developmental theory (30), successful self-exploration of a career path contributes to healthy personal development. However, COVID-19 outbreak, occupational safety, and decreased job expectations for students may challenge students' personal development (31).

### Intolerance of uncertainty and future career anxiety

Uncertainty rains down throughout COVID-19, and IoU nudges people into mental health difficulties involving fear, depression, and anxiety (32). During COVID-19, uncertainty unsettles the general population, but also hurts potential college graduates who are looking for jobs. In their early 20s, they are transitioning from students to workers, making career decisions, and establishing job market commitments (33). However, COVID-19 may undermine the economy and restrict employment, making it difficult for those preparing to graduate from college to determine their job prospects and career opportunities. Thus, students may feel anxious and worried as they explore career options. FCA is the mental stress that people feel when they worry about their future career paths (34). When COVID-19 spreads and labor markets shrink, FCA among college students becomes a critical concern for educators and researchers (23, 35). Grupe and Nitschke (36) pointed out that a steady environment creates a sense of security, whereas volatile circumstances incubate anxiety. Past studies have also emphasized the strong link between anxiety and the need for control (37–40). People have cognitive control when they can predict the presence of a threat and assess their ability to respond (41). However, when people perceive uncertainty and unpredictability in the present, it would make them feel anxious about the future (39, 42). With a similar logic, IoU is tightly wired to FCA as students with higher IoU doubt their ability to eliminate threats in their job search (43). Students with a higher IoU have a more difficult time transitioning from student to work than students with a lower IoU because they are less likely to act in the job market, thereby worsening their FCA (44, 45). Unstable labor markets and fragile economies erode occupational uncertainty and security during COVID-19 (46, 47). Cross-cultural evidence suggested that IoU leads to FCA during COVID-19. In the United States, college students with intense IoU during COVID-19 report less job readiness, which results in FCA (48). Back to China, Chen and Zeng (43) proposed that IoU might lay the groundwork for FCA during COVID-19, not least for university students who anchor their career objectives. Li et al. (7) demonstrated that IoU and FCA are moving in a positive direction among the Chinese. On the contrary, employment is particularly meaningful in Confucian culture for bringing honor to the family, and students are worried about whether their future jobs will meet family needs (49, 50). Aside from social pressure, previous studies have also shown that students who carry higher IoU are more vulnerable to FCA because IoU has a detrimental impact on cognitive resources and distracts them from coping with FCA (43).

### Intolerance of uncertainty, fear of COVID-19, depression, and future career anxiety

Emotions are complex sensory states that affect psychological states and primarily influence thinking and action (51). The cognitive–motivational–relational theory of emotion demonstrates that individuals first label the external stimulus and both personal experiences and social culture influence labeling (52). Feelings are the result of labeling and have an impact on changes in mental states (53, 54). So far, dense IoU impedes normal emotional functioning (55). Individuals with dense IoU may perceive warning signals as overly sensitive and generalized (56). Thus, dealing with an unpredictable infection may induce fear; individuals commonly sound alarms and overuse cognitive resources to cope with all perceived threats (57). COVID-19 is an unpredictable health crisis that harms not only an individual's mental or physical health but also their career development, spreading fear among forthcoming university graduate students (1, 58). FoC is an alerted oriented emotional response to COVID-19 that threatens an individual's life, net health, social connections, and economic activities (59). COVID-19 fluctuates, and people are unsure when the virus will vanish; they yearn for pre-pandemic life, which fuels the FoC (60, 61). For instance, Millroth and Frey (2020) found a positive correlation between IoU and FoC, with deeper IoU predicting stronger FoC within a Swedish sample. Similarly, Satici et al. (21) also discovered that IoU remained positively affiliated with FoC in Turkey.

Furthermore, FoC predicted FCA in a positive way. During a pandemic, people first worry about their health, but they gradually fear a pandemic-induced economic slump, making individuals feel insecure about their current jobs and future career paths (22). The International Monetary Fund (IMF) reported during COVID-19 that the impending recession would lead to a multi-layered financial collapse caused by declining export growth and lower product market prices in countless countries (62–66). Alici and Copur (67) also demonstrated that Turkish nursing students suffer from FCA because they fear that pandemic will affect employment rates. Thus, FoC is mounting with FCA positively in a few developing countries (32, 68). However, future exploration in a Confucian developing state such as China is worthwhile. Given that Confucian culture emphasizes personal behaviors that affect family reputation and that students' careers are linked to family achievement, the pandemic may pose a formidable threat to the careers of Chinese students (69). Asian values include high regard for family honor and a desire for careers to match family expectations (70).

In addition to anxiety, individuals are susceptible to depression when the external environment is varying (71). IoU contributes to depressive feelings, while intense FCA is positive relative to severe worry and rumination, but the underlying mechanisms remain puzzling (54, 61, 72). Depression is defined as intense negative feelings that let people's cognition, emotions, and behaviors to become dysfunctional, manifesting as persistent sadness, helplessness, and diminished desire (28, 73). According to the helplessness–hopelessness model, IoU and hopelessness are owing to future depression (74, 75). The model adds this approach to the cognitive process, explaining that IoU leads to a sense of instability in allergy and eventually to a chain of depression (76). Dupuy and Ladouceur (76) revealed that IoU triggers otiose concerns and people who have depression; they prefer to experience a disaster rather than uncertainty. Whenever fear is condensed enough to dig people into dysfunction, they might feel depressed (77).

Previous studies have noted that FoC impairs mental health and is related to depressive symptoms (78, 79). Erbiçer et al. (77) outlined a positive association between FoC and depression. Ashraf et al. (80) suggested that FoC connects positively with situational depression when an epidemic hinders social activities and reduces wellbeing in Pakistan. In China, Huang and Zhao (81) discovered that FoC is positively linked with depressive mood as people were concerned about the controllability of the epidemic. Furthermore, previous research has found that depression frequently coexists with anxiety (82). In various countries, depression is confluent with anxiety risk, including FCA (76, 83). People with more severe depressive symptoms exert indecision about their future occupations and foster FCA (61, 83). In the outbreak, Chowdhury et al. (84) concluded that depression and FCA are related in a positive way among Bangladeshi students. Thus, students who are distressed about employment opportunities are upset about economic stagnation and potential career advancement (84).

Whenever fear is condensed enough to dig people into dysfunction, they might feel depressed (77). Previous research has suggested that FoC is blamed for mental health issues such as depression and that people with higher FoC levels are more prone to suffer from depression (85). Sakib et al. (86) concluded that FoC was positively associated with depression in the general population and among health professionals, especially women and singles in Bangladesh. Yalçn et al. (79) also demonstrated that FoC correlates with depression positively among Turkish students. Most of the past research on IoU and FCA has focused on areas with a strong influence of Abrahamic religions, and relatively little research has been done on Confucian collectivist culture. In the United States, where Christianity is the dominant religion, and in Turkey, where Islam is the dominant religion, the focus of Abraham's religion aligns with the aspirations of high power (87, 88). Thus, future research into Confucian culture, which values family obligations, is warranted.

### The present study

The current study aims to explore the role of depression and FoC in the association between IoU and FCA among Chinese undergraduate students during the COVID-19 pandemic. Few studies have elucidated the relationship between IoU and future career concerns. However, most past studies have obtained strong evidence from South Asia, the Middle East, or Western countries. Considering Confucianism and the one-child policy, Chinese millennials may face more career-related pressure than their elders, as high-flying jobs serve not only as anchors in their lives but also polish family honor (89, 90). The enormous difficulty, however, is that the tumultuous work environment is changing with each passing day, yet the pandemic has not removed social expectations. Besides, even though several studies have been conducted with Chinese university students, they have focused on the buffering effects of FCA, such as resilience, rather than providing insight into the knock-on effects of negative emotions.

Thus, it is critical to comprehend FCA within the context of Confucian cultural heritage and unique population policy during the pandemic. Besides, FCA is a complex process threatened by a variety of negative resources, including anxiety, fear, and external stress reactions such as FoC. This study examines negative affective chains, depression, and FoC from a cultural and age perspective, aiming to analyze how these variables influence the relationship between IoU and FCA among Chinese college students.

To evaluate the relationships among the above variables, the following hypotheses were proposed:

Hypothesis 1 (H1): IoU will be positively correlated with FCA;
Hypothesis 2 (H2): FoC will mediate the association between IoU and FCA;
Hypothesis 3 (H3): Depression will mediate the association between IoU and FCA;
Hypothesis 4 (H4): The association between IoU and FCA will be mediated through the chain mediating effects of FoC and depression.

**Figure 1:**
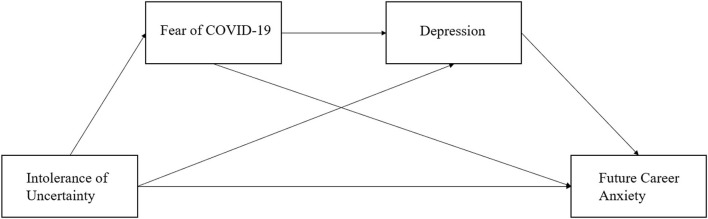
Conceptual model.

The conceptual model of hypotheses is shown in Figure 1.

## Methods

### Participants and procedures

Regarding the quarantine policies and social distance needs during the COVID-19 pandemic period, an online self-administered survey was applied to collect data for this study. Online posters were used to distribute invitations to participate in the study, along with details about the survey link, the objectives of this study, confidentiality policies, and contact information for the researchers. All participation in this study was anonymous and voluntary. This study involved 1,947 participants, with 1,919 valid response (98.6%; mean age = 19.34 ± 1.55 years), including 802 males (41.5%), 1,111 females (51.3%), and 6 unwilling to report or other genders (0.3%) from 11 universities in eight Chinese cities. The data collection procedure was conducted from October 2021 to November 2021, and all valid participants were undergraduate students currently studying at Chinese universities, and all ineligible participants (non-undergraduate participants) or subjects in completed responses were excluded from the data analysis.

### Measures

#### Demographic information

Participants were required to provide their age, gender (1 = males, 2 = females, and 3 = unwilling to report or other genders), and current institutions.

#### Intolerance of Uncertainty (IoU)

Intolerance of uncertainty was assessed by using the 12-item Intolerance of Uncertainty Scale (IUS-12) developed by Carleton et al. (91). This scale is a shortened version of the 27-item Intolerance of Uncertainty Scale (IUS-21) (92) used to describe negative attitudes and reactions to uncertainty, with each item rated on a five-point Likert scale (from 1 = not at all characteristic of me to 5 = entirely characteristic of me) with a higher mean score indicating a stronger reaction to uncertainty (e.g., “When I am uncertain I can't function well”). Wu et al. (93) validated IUS-12 in Chinese content of the IUS-12 with a Cronbach's alpha of 0.93 for the IUS-12.

#### Fear of COVID-19 (FoC)

This study measured FoC using the Fear of the COVID-19 Scale (FCV-19S) (94). The FCV-19S contains seven items that measured the level of FoC in two factors, namely, physical response of fear and fear thinking; each item was scored on a five-point scale (1 = disagree; 5 = completely agree), with a higher mean score reflecting a higher level of FoC (e.g., “I am most afraid of the COVID-19). The FCV-19S was validated in well-used Chinese content by Chi et al. (95), with a Cronbach's alpha of 0.89 for the FCV-19.

#### Depression

Depression was assessed using the Patient Health Questionnaire (PHQ-9) (96), which contains nine items that measure the presence and status of depression; each item was rated on a four-point scale (from 0 = not at all to 3 = nearly every day), with a higher score reflecting a higher level of depressive symptoms (e.g., “Little interest or pleasure in doing things). The previous study has validated PHQ-9 in different contents, including Chinese content (97). The Cronbach's alpha of the PHQ-9 was 0.94.

#### Future career anxiety (FCA)

Future career anxiety was assessed using the five-item Future Career-related Anxiety Scale developed by Mahmud et al. (22), an adapted version of the Career Anxiety Scale (33) to measure anxiety toward future careers. Each item was rated on a four-point scale (from 1 = strongly disagree to 4 = strongly agree), with a higher mean score indicating a stronger future career-related anxiety (e.g., “I worry about future employment because of a potential economic recession due to the outbreak of COVID-19). The Cronbach's alpha of the Future Career-related Anxiety Scale was 0.96.

### Statistical analysis

Statistical Package of Social Science software version 26 (SPSS 26.0) was used for data analysis. Descriptive analyses were used to analyze demographic variables. To explore the bivariate correlations among IoU, FoC, depression, and FCA, Pearson's correlation was calculated. The mediating roles of FoC and depression were tested using Model 6 in SPSS PROCESS macro version 3.5.3 (98); 95% confidence intervals of the indirect effects were calculated on resampling of 5,000 bootstrap estimates, and the mediating effect was significant at *p*<*0.05* when the confidence interval did not include zero.

## Results

### Bivariate correlations between study variables

Descriptive statistics and correlations for all variables are given in Table 1. IoU was positively correlated with FoC (*r* = 0.61, *p* < 0.001), depression (*r* = 0.52, *p* < 0.001), and FCA (*r* = 0.51, *p* < 0.001). Besides, both FoC (*r* = 0.46, p < 0.001) and depression (*r* = 0.62, p < 0.001) were positively correlated with FCA. Meanwhile, FoC also showed a significant positive correlation with depression (*r* = 0.48, *p* < 0.001).

**Table 1:** Correlations between variables (*N* = 1,919).

	***M***	**SD**	**1**	**2**	**3**	**4**
1. Intolerance of Uncertainty	2.69	0.79	1			
2. Fear of COVID-19	2.37	0.84	0.51^***^	1		
3. Depression	4.55	4.30	0.46^***^	0.61^***^	1	
4. Future Career Anxiety	2.58	1.02	0.62^***^	0.52^***^	0.48^***^	1

^***^p < 0.001.

### Chain mediation model analysis

After controlling for gender and age, the mediating effect of FoC and depression in the relationship between IoU and FCA was analyzed, as given in Table 2.

**Table 2:** Regression analysis of relationship between Fear of COVID-19 and Depression with mediation analyses (*N* = 1,919).

**Regression equation**		**Fitting index**			**Significance**		
**Result variable**	**Predictor variable**	***R***	***R* ^2^**	***F***	**Coeff**	**Standardized** **Coeff**.	***t***
Fear of COVID-19		0.525	0.275	242.349^***^	0.509		2.321^*^
	Gender				0.209	0.125	6.402^***^
	Age				0.002	0.004	0.228
	Intolerance of Uncertainty				0.548	0.515	26.386^***^
Depression		0.639	0.408	330.020^***^	−1.051		−6.177^***^
	Gender				−0.041	−0.028	−1.588
	Age				0.0182	0.039	2.228^*^
	Intolerance of Uncertainty				0.177	0.194	9.442^***^
	Fear of COVID-19				0.442	0.516	24.989^***^
Future Career Anxiety		0.626	0.391	410.172^***^	−0.679		−2.758^**^
	Gender				0.073	0.035	1.979^*^
	Age				0.051	0.077	4.281^**^
	Intolerance of Uncertainty				0.805	0.617	34.54^***^
Future Career Anxiety		0.677	0.458	323.513^***^	−0.616		−2.623^***^
	Gender				0.012	0.006	0.035
	Age				0.045	0.069	0.011^***^
	Intolerance of Uncertainty				0.584	0.448	22.246^***^
	Fear of COVID-19				0.234	0.191	8.385^***^
	Depression				0.219	0.154	7.032^***^

^*^p < 0.05,

^**^p < 0.01,

^***^p < 0.001.

When gender and age were included in the regression model as four control variables, the results showed that IoU was a significant positive predictor of FCA (*B* = 0.617, *p* < 0.001), and H1 was supported.

Moreover, both FoC (*B* = 0.191, *p* < 0.001) and depression (*B* = 0.154, *p* < 0.001) were significant positive predictors of FCA, and the direct path from IoU to FCA (*B* = 0.516, *p* < 0.001) was significant. Meanwhile, the direct effect of IoU on FoC (*B* = 0.515, *p* < 0.001) and depression (*B* = 0.194, *p* < 0.001) was significant.

**Figure 2:**
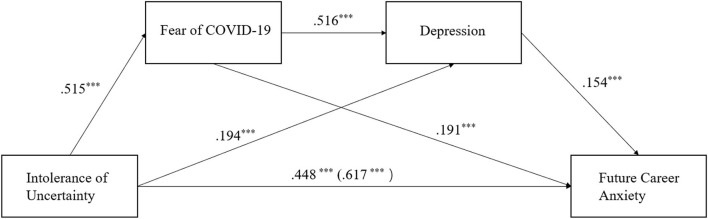
The chain mediation model. Note. ***p < 0.001.

The mediating effects of FoC and depression between IoU and FCA are given in Table 2, and Figure 2 shows a model of the cascading mediating effects between IoU and FCA. FoC and depression were significant mediators between IoU and FCA (β = 0.220, *SE* = 0.016, 95% *CI* = 0.189 to.252). All three indirect paths in the mediation model were significant (Table 3): path 1(H2) - IoU → FoC → FCA (β = 0.128, *SE* = 0.017, 95% *CI* = 0.095 to.162), path 2 (H3) - IoU → depression → FCA (β = 0.039, *SE* = 0.007, 95% *CI* = 0.027 to.052), path 3 (H4) - IoU → FoC → depression → FCA (β = 0.053, *SE* = 0.008, 95% *CI* = 0.038 to.069). As the 95% *CI* in all those paths did not contain a value of 0, the results confirmed that H2, H3, and H4 were all supported.

**Table 3:** Fear of COVID-19 and depression in the mediation effect analysis (N = 1,919).

	**Indirect effects**	**Boot SE**	**Boot LLCI**	**Boot LLCI**
Total indirect effect	0.220	0.016	0.189	0.252
Indirect effect 1	0.128	0.017	0.095	0.162
Indirect effect 2	0.039	0.007	0.027	0.052
Indirect effect 3	0.053	0.008	0.038	0.069

Indirect effect 1 (H2): intolerance of uncertainty → fear of COVID-19 → future career anxiety;

Indirect effect 2 (H3): intolerance of uncertainty → depression → future career anxiety; and

Indirect effect 3 (H4): intolerance of uncertainty → fear of COVID-19 → depression → future career anxiety.

## Discussion and conclusion

### Discussion

This study aimed to investigate whether a sequential mediation forecasts stress-induced factors and to estimate the contribution of these factors to the COVID-19 pandemic and FCA. Specifically, this study explored the fear and depression experienced by individuals during the COVID-19 pandemic and allows us to reveal potential mechanisms between their IoU and FCA levels. The results from a sequential mediation model indicated that IoU led to higher FoC, which increased levels of depressive symptoms and increased FCA. Explicitly, the results implied a significant positive relationship between IoU and FCA among Chinese students. Furthermore, FoC, depression, and the chain mediating effect of FoC and depression may act as mediating factors, indirectly affecting the relationships between IoU and FCA.

The findings of the bivariate correlations indicated that the direct effects of IoU on FCA were significant and positive, implying that IoU gives rise to FCA among undergraduate students during the COVID-19 epidemic in China, and that IoU is a breeding ground for adolescent anxiety symptoms associated with future career development, which brings them into correspondence with previous findings (23, 35). Drawing on He and Yu (23), which examined the career adjustment characteristics of 1,160 recent Chinese college graduates during COVID-19 in 2021, career adaptability was found to be affiliated with less IoU and anxiety sensitivity. Hite and McDonald (35) outlined how uncertainty shakes up job opportunities, reflected in how it shapes contracted careers, but in the end, students have no choice but to deal with these challenges. According to the career construction theory, Brown and Lent (99) assume that youth's internal characteristics and external environmental factors would contribute to career development together. However, uncertainties make it hard for young people to decide what kind of person they want to be and what kind of work they want to do (like self-employment), which makes them worry even more about their future careers (22). Anxiety, on the contrary, is fired by the potential danger of spiraling, out of control (36). Those who fear the uncertainty of being drawn to negative cues, whether about life or work, trigger increasing symptoms of anxiety. In addition, young individuals with greater IoU have negative filters toward the world and they perceive themselves as having fewer resources to deal with unstable environments, which can diminish their self-efficacy to combat FCA (92, 100, 101).

Moreover, the results displayed that FoC significantly mediated the association between IoU and FCA. This result was a mirror of that of previous studies that found IoU in youth can increase their risk of FoC and potential occupational anxiety [e.g., 15, 72]. Pak et al. (102) conducted an online cross-sectional survey of 362 participants in Turkey and discovered that FoC has developed as a result of IoU. Satici et al. (21) investigated 1,772 Turkish individuals and suggested that FoC serially mediated the association between IoU and mental wellbeing. This may be explained by the fear generalization mode (103). Considering the above models, they suggested that people feel fear in the face of uncertainty, which depletes their decision-making capacity and drives them to feel more negative emotions, such as anxiety. Besides, high levels of uncertainty may discount individuals' cognitive functioning and exacerbate FoC as they become emotionally depressed while coping (103). Moreover, this study's result also confirmed that IoU exerted a significant indirect effect on depression *via* FoC, confirming previous findings (102, 104). Individuals with a high IoU were more likely to perceive the pandemic as a threat, and their anxiety levels skyrocketed, partly due to insufficient governmental support (102). Employees have been floundering in an uncertain work environment and have been enduring job insecurity since the COVID-19 outbreak (35, 46). Living in a perilous environment, Chinese college students have become befuddled and doubted their employment opportunities (23).

Consistent with past research, the findings also suggested that depression mediated the association between IoU and FCA and IoU was related to depression and that young adults with depressive symptoms have higher chances of developing FCA (22, 102). The helplessness–hopelessness model suggests that depression is a psychological reaction driven by IoU characteristics and the hopelessness of future expectations (74). Besides, IoU triggers unnecessary individual concern and rumination, which can lead to depression (76). Depressive symptoms may affect individuals' cognitive functioning, with individuals with higher levels of depressive symptoms having low cognitive functioning and worrying about future careers, which can propagate worse FCA (83).

Moreover, this sequential mediation model led to a positive association between FoC and depression, with a chain mediating effect between FoC and depression mediating the relationship between IoU and FCA. These results were in line with former findings that individuals with higher FoC also exhibit severe depressive symptoms (11, 78). Bakioglu et al. (32) indicated that high levels of FoC incline to worsen depressive symptoms as they are fenced in by high infectivity, dangerous outcomes, social distance, and confinement together. In addition, as is the nature of all pandemics, the COVID-19 pandemic brings uncertainty drifting around every corner, neither finances, health, nor daily social activities. Such a dystopian environment steals positive emotions from the population (102).

### Implications

The findings of this study have vital implications for the mental health of college students. First, past research has focused on how COVID-19-related stress predicts their current academic anxiety. However, this study concentrated on pandemic-induced unpleasant feelings that might influence future occupational anxiety (105, 106). Second, rather than concentrating on regions affected by Abraham's religion, such as the United States and the Middle East (48, 102, 107), this study examined the relationship between pandemic-induced negative emotions and FCA among Chinese college students raised in a Confucian culture whose personal careers were highly bonded to their family reputation (49, 50). This study showed these mental difficulties impact their lives: The higher the IoU among college graduates, the more FCA climbed. Students with stronger IoU have limited cognitive resources to overcome the FCA. An unstable environment prevents people from effectively seeking jobs. This study identifies the former theory in the context of the pandemic and contributes to understanding these complex factors by training college students to have a healthy attitude toward future employment, which is indispensable for their current developmental stage. Uncovering how FoC impacts FCA can provide information to college occupational professionals to assist students' employment preparation. The adverse effects of FoC are comparable to the symptoms of general anxiety (21, 102). Students may be unaware that their nervousness or concerns about their future careers result from their dread of the epidemic. Therefore, understating how FoC affects FCA has practical implications. Professionals in school mental health may provide social or psychological assistance to students with depressive symptoms and pandemic anxiety. The college occupational counselor should advise students that the pandemic is not as fateful as it may seem and that it will not hinder the labor market in the long run.

### Limitations

This study has some limitations that need to be declared. First, this study used a cross-sectional design and was unable to reveal the long-term stress caused by the pandemic and its effects on FCA. Second, this study did not categorize the participants' grade levels and majors, but students in upper grades or humanities may face greater job search stress. Third, self-reported questionnaires may include autobiographical memory bias, which tends to downplay their difficult experiences (108). Finally, geographic differences were evident. Participants from developed areas such as Suzhou may have different FCA levels than participants from developing regions such as Mudanjiang due to heterogeneous industrial structures and labor demand. Thus, how geographic and economic factors moderate the association between IoU and FCA is an exploratory option for the future.

### Conclusion

In conclusion, this study demonstrated the mediating effect of FoC and depression on the relationship between IoU and FCA among Chinese undergraduate students during the COVID-19 pandemic; thereby, the finding provided empirical evidence to confirm not only a positive relationship between IoU and FCA but also the chain mediation effect of FoC and depression. Therefore, it is important to provide information to motivate schools to offer career opportunity workshops and, most importantly, mental health support to guide students to effectively cope with uncertainty and overcome the stress associated with COVID-19.

## Data availability statement

The raw data supporting the conclusions of this article will be made available by the authors, without undue reservation.

## Ethics statement

The studies involving human participants were reviewed and approved by Suzhou University of Science and Technology. The patients/participants provided their written informed consent to participate in this study.

## Author contributions

TZ and DG designed the research. TZ, DG, YBai, HL, and YH completed the manuscript writing. XC collected and analyzed the data. RW, YBao, and XC reviewed and edited the manuscript. All authors contributed to the article and approved the submitted version.

## Funding

The present study was funded by National Social Science Foundation of China: Youth Project (Grant No. 19CGJ005). This study was also supported by Suzhou University of Science and Technology.

## Conflict of interest

The authors declare that the research was conducted in the absence of any commercial or financial relationships that could be construed as a potential conflict of interest.

## Publisher's note

All claims expressed in this article are solely those of the authors and do not necessarily represent those of their affiliated organizations, or those of the publisher, the editors and the reviewers. Any product that may be evaluated in this article, or claim that may be made by its manufacturer, is not guaranteed or endorsed by the publisher.
